# γ-Aminobutyric acid may directly or indirectly regulate Arabidopsis ALMT9

**DOI:** 10.1093/plphys/kiac399

**Published:** 2022-09-01

**Authors:** Matthew Gilliham, Bo Xu

**Affiliations:** Waite Research Institute and School of Agriculture, Food and Wine, University of Adelaide, Glen Osmond, South Australia 5064, Australia; Waite Research Institute and School of Agriculture, Food and Wine, University of Adelaide, Glen Osmond, South Australia 5064, Australia

## Abstract

The mechanism by which GABA regulates stomatal pore aperture and anion transport activity of ALUMINUM-ACTIVATED MALATE TRANSPORTER 9 is debated.

Dear Editor,

γ-Aminobutyric acid (GABA) is a plant metabolite that oscillates in concentration over diel cycles (Espinoza et al., 2010) and accumulates when plants are subjected to various abiotic and biotic stresses (Kinnersley and Turano, 2000). Evidence that GABA acts as an endogenous signal to modify plant physiology has been building over the past decades. The discovery that GABA can modify anion current activity of ALUMINUM-ACTIVATED MALATE TRANSPORTERS (ALMTs) (Ramesh et al., 2015; Domingos et al., 2019; Long et al., 2019) provides a mode of action by which GABA could act as a membrane potential-based signal that links plant metabolic status with multiple physiological outputs (Gilliham and Tyerman, 2016). These processes include aluminum tolerance, root growth, pollen tube growth, and stomatal pore regulation, all of which are modulated following modification of GABA metabolism or exogenous GABA supply and are known to involve ALMTs (Ramesh et al., 2017). While evidence suggests that GABA can directly alter the anion transport activity of a number of ALMT (Ramesh et al., 2015), this work has mostly focused on *Triticum aestivum* (wheat) ALMT1, which has also been proposed to transport GABA (Ramesh et al., 2018), and GABA binding has not been proven (Xu et al., 2021b). So, the nature of the GABA–ALMT interaction is still not fully resolved.

Recently, Xu et al. (2021a) provided multiple lines of genetic and physiological evidence that GABA reduces stomatal opening to improve water use efficiency, and that in Arabidopsis (*Arabidopsis thaliana*) this modulation likely depends upon tonoplast-localized AtALMT9. In a follow-up study, Jaślan and De Angeli (2022) clearly show no impact of GABA on anion currents carried by AtALMT9 transiently expressed in *Nicotiana benthamiana* mesophyll cells, which was presented as proof, as highlighted by the title, that GABA “does not directly inhibit AtALMT9.” While we welcome the study and do not question the data, such a result allows for such a conclusion to be drawn only in the heterologous system used for testing. Jaślan and De Angeli (2022) transiently expressed the Arabidopsis protein AtALMT9 fused to green fluorescent protein (GFP) using *Agrobacterium*-based infection of *N. benthamiana* leaves, followed by enzymatic digestion to remove the cell walls and release protoplasts and osmotic shock to burst protoplasts and isolate vacuoles. These vacuolar membranes were then subjected to patch clamp electrophysiology using highly artificial solutions that diluted vacuolar contents, were devoid of standard cytosolic factors, and were dominated by Bis–Tris propane to reduce native currents (De Angeli et al., 2013). Results from the heterologous system used by Jaślan and De Angeli (2022) have been used previously to suggest regulators when an experimental interaction was observed (e.g. Zhang et al., 2013; Figure 1), but no definitive statements regarding in planta function can be made, especially with respect to negative results. Therefore, the results of Jaślan and De Angeli (2022) can be used to legitimately cast doubt on whether direct GABA regulation occurs for AtALMT9, but reliance on this single reductionist and heterologous experimental approach without confirmation via other approaches, including in planta assays, precludes irrefutable extrapolation of negative results to the native system. Therefore, we suggest the question of how GABA regulates AtALMT9 remains very much open.

**Figure 1:**
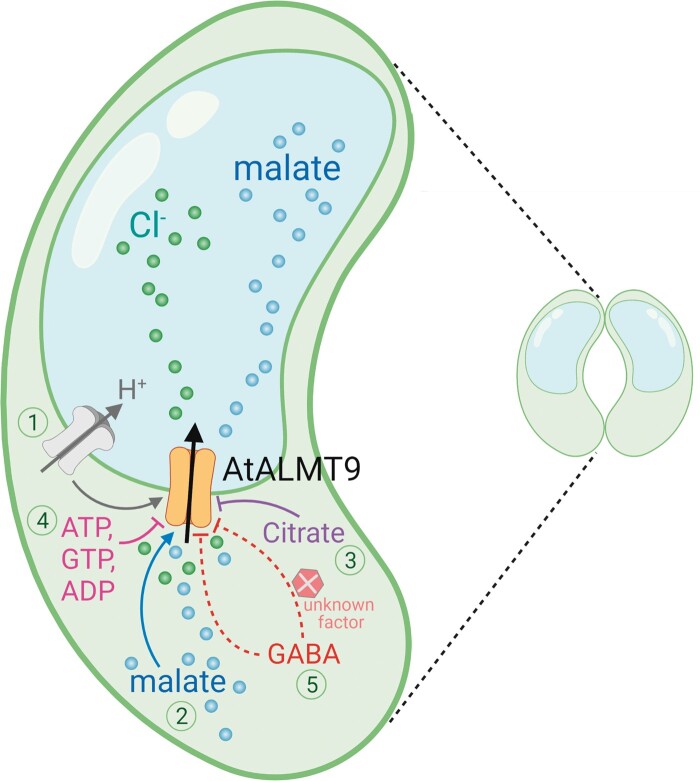
Summary of factors currently proposed to regulate AtALMT9 activity in stomatal guard cells. (1) Voltage driven by the tonoplast proton (H^+^) pump to generate electrical gradients as a driving force for AtALMT9-mediated chloride (Cl^−^) and malate uptake (De Angeli et al., 2013); (2) malate stimulated Cl^−^ uptake (De Angeli et al., 2013); (3) cytosolic citrate suppression of AtALMT9-mediated malate transport (Zhang et al., 2013); (4) cytosolic nucleotides inhibition of AtALMT9-mediated Cl^−^ and malate uptake, including adenosine 5′-triphosphate (ATP), guanosine-5′-triphosphate (GTP), and adenosine di-phosphate (ADP) (Zhang et al., 2014); and (5) AtALMT9-dependent GABA regulation of stomatal opening (Xu et al., 2021a). As stated by Xu et al. (2021a), AtALMT9 may be indirectly regulated by GABA (Jaślan and De Angeli, 2022), possibly via an unknown intermediate (*x*), or directly (as hypothesized for other ALMTs) (e.g. Long et al., 2019). Protein phosphorylation also appears critical in regulating and stabilizing ALMT transport activity (e.g. Ligaba et al., 2013; Eisenach et al., 2017; Qin et al., 2022), but not yet for AtALMT9, so is not shown on the diagram. All listed factors may work together to coordinate AtALMT9 regulation and contribute to regulation of stomatal opening and aperture. It is noted that citrate, ATP, ADP, and GTP all have roles in stomatal regulation (Fairley-Grenot and Assmann, 1991; Wang et al., 2001; Chen et al., 2017; Su et al., 2017; Lim et al., 2022); however, the impact of their regulation on AtALMT9 in planta has not been confirmed. The graph is created using Biorender.

Reasons for this are manifold and include, but are not limited to, not knowing, or defining: whether the Arabidopsis protein folds or functions correctly in *N. benthamiana*; whether the GFP tag interferes with function; and, if AtALMT9 performs in mesophyll cell membranes of *N. benthamiana* as it does in Arabidopsis guard cells, as clearly the same suite of regulatory and interacting factors would not be present. GFP tags can interfere with protein function, including other ALMTs (Mumm et al., 2013), and while protein tagging is regularly used for experimental assays it can complicate interpretations. For instance, it should be noted that in the study of Xu et al. (2021a), a GFP-tagged AtALMT9 is used to complement *almt9*, but as the wild-type and point mutant-containing plants show differential GABA sensitivity, it provides some reassurance of AtALMT9 retaining function in Arabidopsis. However, non-tagged point mutants created with gene editing or traditional mutagenesis would be a useful tool for additional experiments. Further, the patch clamping of Arabidopsis proteins in Arabidopsis membranes, although still suffering from a dilution of cellular factors and the presence of currents that can mask ALMT activity (De Angeli et al., 2013), would be a step closer to the native system and has previously been used to characterize AtALMT9 in Arabidopsis mesophyll and AtALMT6 in guard cells (Kovermann et al., 2007; Meyer et al., 2011; De Angeli et al., 2013). Therefore, this seems a sensible next step to further investigate the mechanism by which GABA regulates stomatal aperture.

Due to the above outlined uncertainties brought by heterologous systems, and their unsuccessful attempts at electrophysiological characterization of AtALMT9, Xu et al. (2021a) state: “Following on from this study … provides the basis for future research areas. Such studies will be able to resolve questions such as ‘whether GABA can act directly on guard cell ALMTs?’, as appears to occur for wheat ALMT1, or ‘whether other signalling intermediates are also involved?’.” Further, in Xu et al. (2021b), a topical review in *Plant Physiology*, the Outstanding Question “Does cytosolic GABA directly interact with ALMT9 and 12 in stomatal regulation?” is posed and the body text states, “An assay is now required to demonstrate whether GABA has a direct effect on ALMT9, as has been demonstrated for TaALMT1 via electrophysiology, or whether GABA acts on ALMT9 via a distinct mechanism.” Jaślan and De Angeli (2022) have provided a step toward discerning between the range of possibilities outlined by Xu et al. (2021a) but surprisingly state that Xu et al. (2021a, 2021b) only propose that GABA directly regulates AtALMT9. Instead, Xu et al. (2021a) detail that GABA modulation of changes in stomatal aperture was dependent upon AtALMT9 (and specific amino acid residues within AtALMT9), but they were unable to resolve whether GABA directly regulates AtALMT9; whether GABA indirectly regulates AtALMT9 through other intermediates; or whether GABA directly or indirectly regulates other proteins to exert its impact on stomatal aperture.

Moving forward, Figure 1 shows all the proposed regulatory factors that have so far been identified experimentally to alter AtALMT9 function, whether using heterologous systems or genetic in planta methods. Importantly, the recent structural resolution of ALMT12 and ALMT1 proteins, both of which form homo-dimeric complexes with the interface of the two subunits forming the pore (Zhang et al., 2013; Gilliham and Hrmova, 2022; Qin et al., 2022; Wang et al., 2022), affords a renewed opportunity to experimentally probe structure–function relations of ALMT. Previous studies prior to ALMT structural resolution correctly identified amino acid residues important for malate permeation within the pore of AtALMT9 (Zhang et al., 2013). New studies could hypothesize the nature of any regulatory interaction through homology modeling, but will need additional structure–function experimental verification to determine the nature of the regulation; for GABA and ALMT, this could differentiate between GABA directly inhibiting anion transport via an allosteric interaction, competition in the pore, or indirect regulation. We are excited by the prospect of future research by multiple groups into the questions surrounding GABA as a signal in plants, including the role of ALMTs, and encourage the use of more sophisticated approaches than those so far deployed to provide definitive answers.

## Notes

B.X. and M.G. wrote this letter.

The author responsible for distribution of materials integral to the findings presented in this article in accordance with the policy described in the Instructions for Authors (https://academic.oup.com/plphys/pages/general-instructions) is: Bo Xu (b.xu@adelaide.edu.au).
